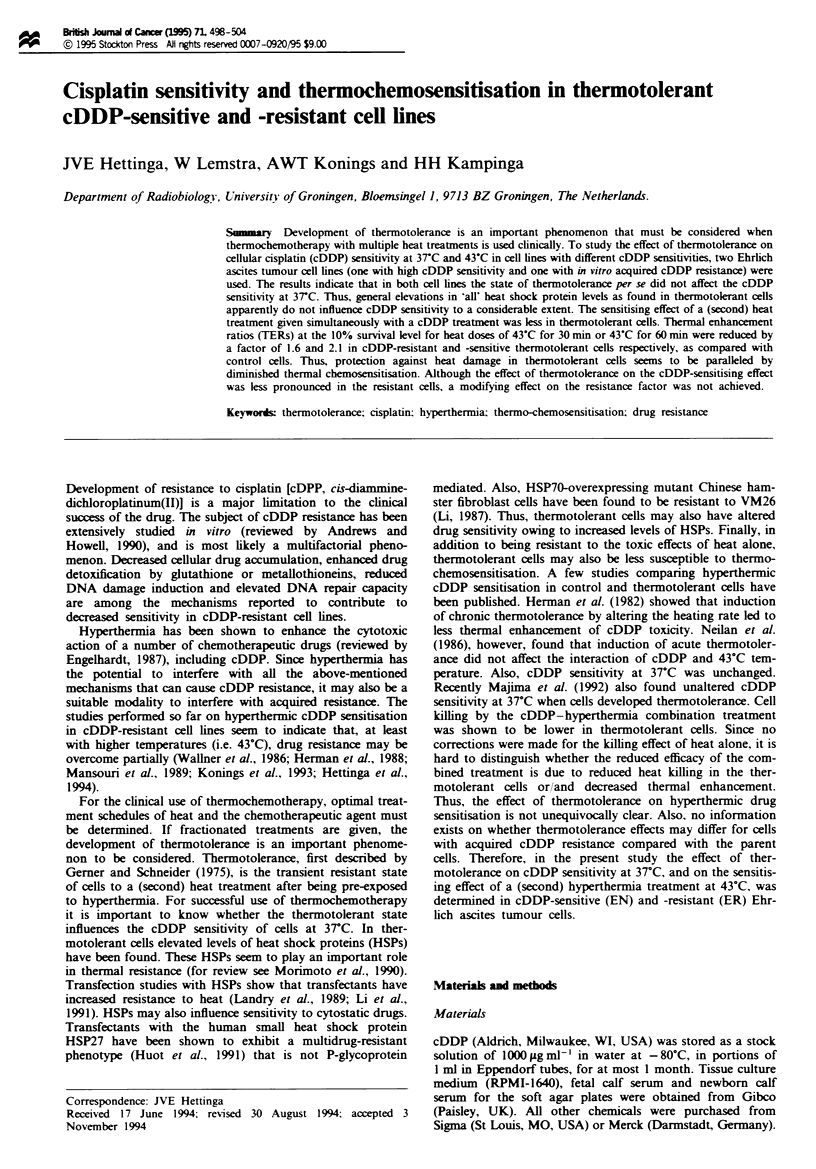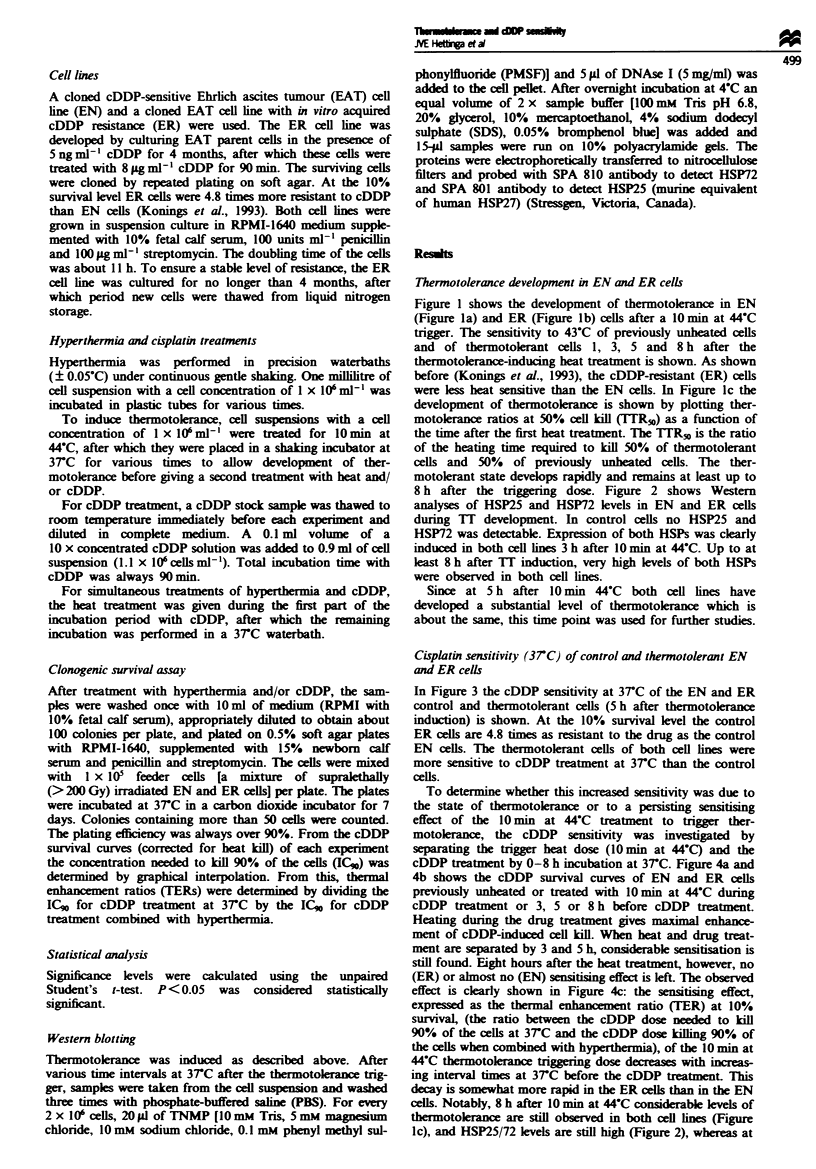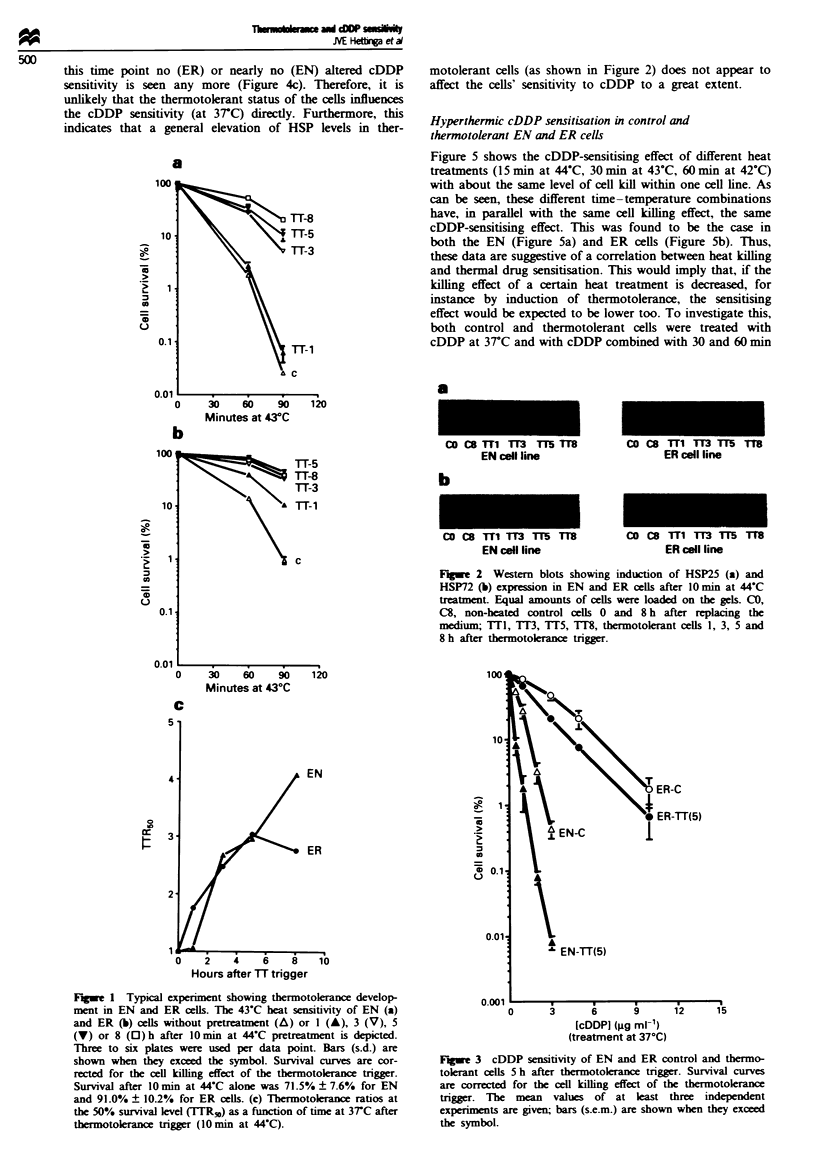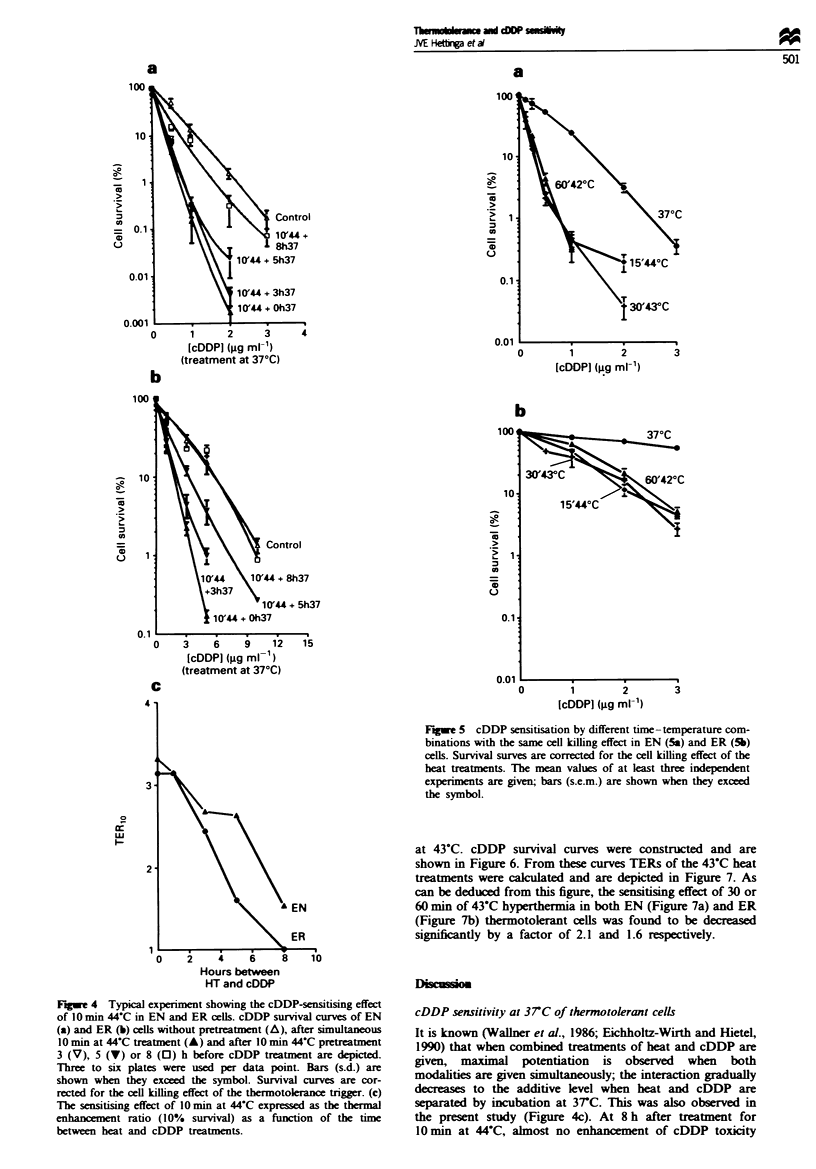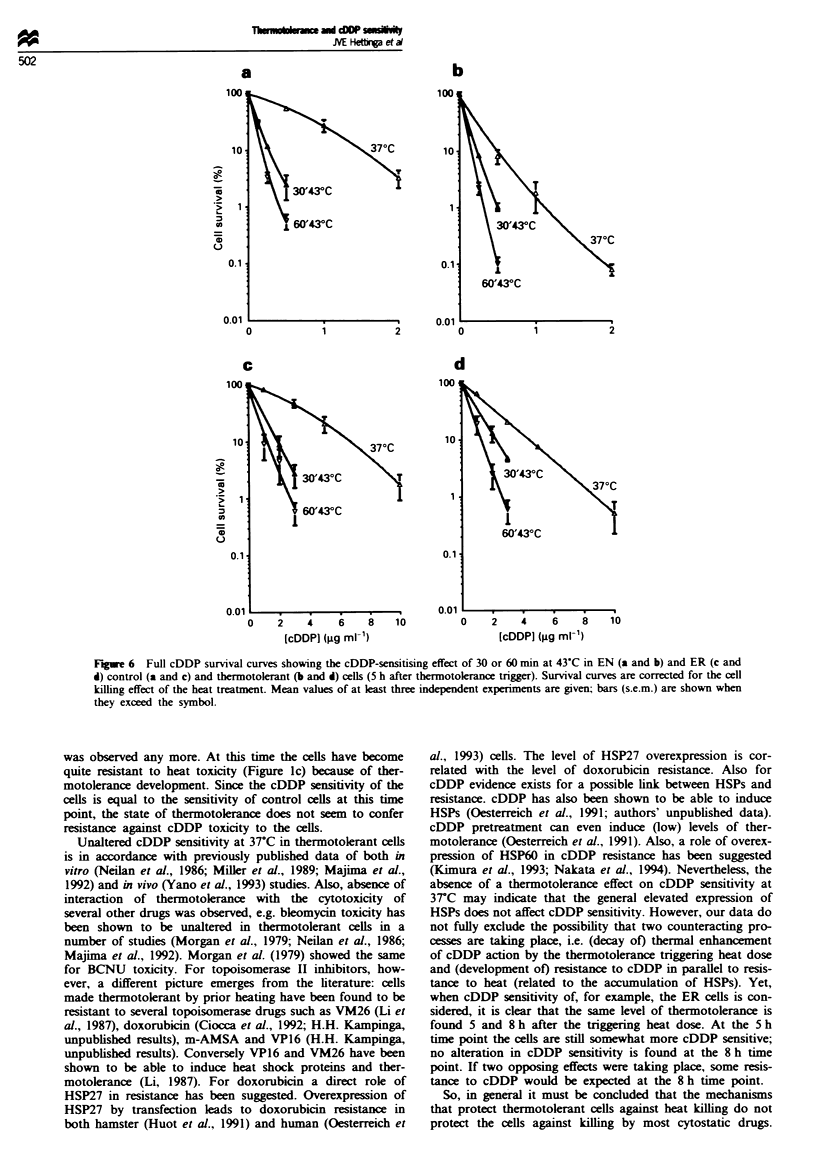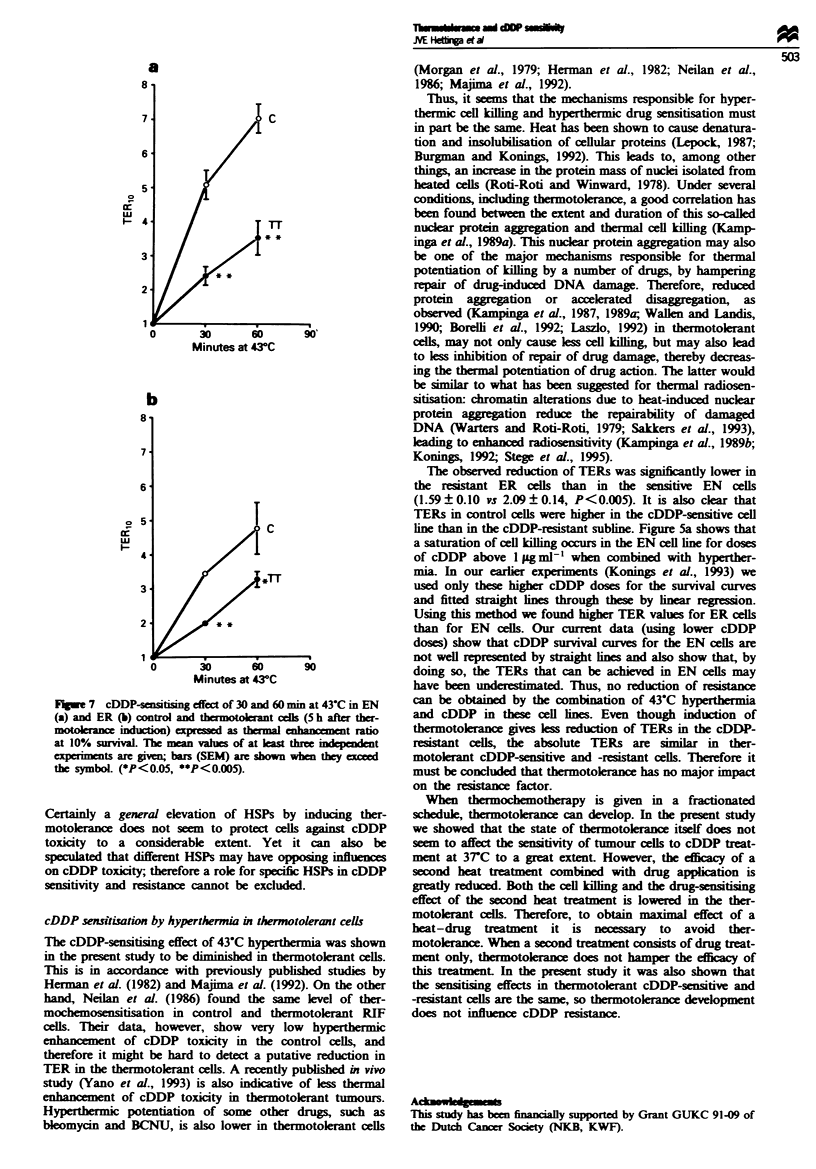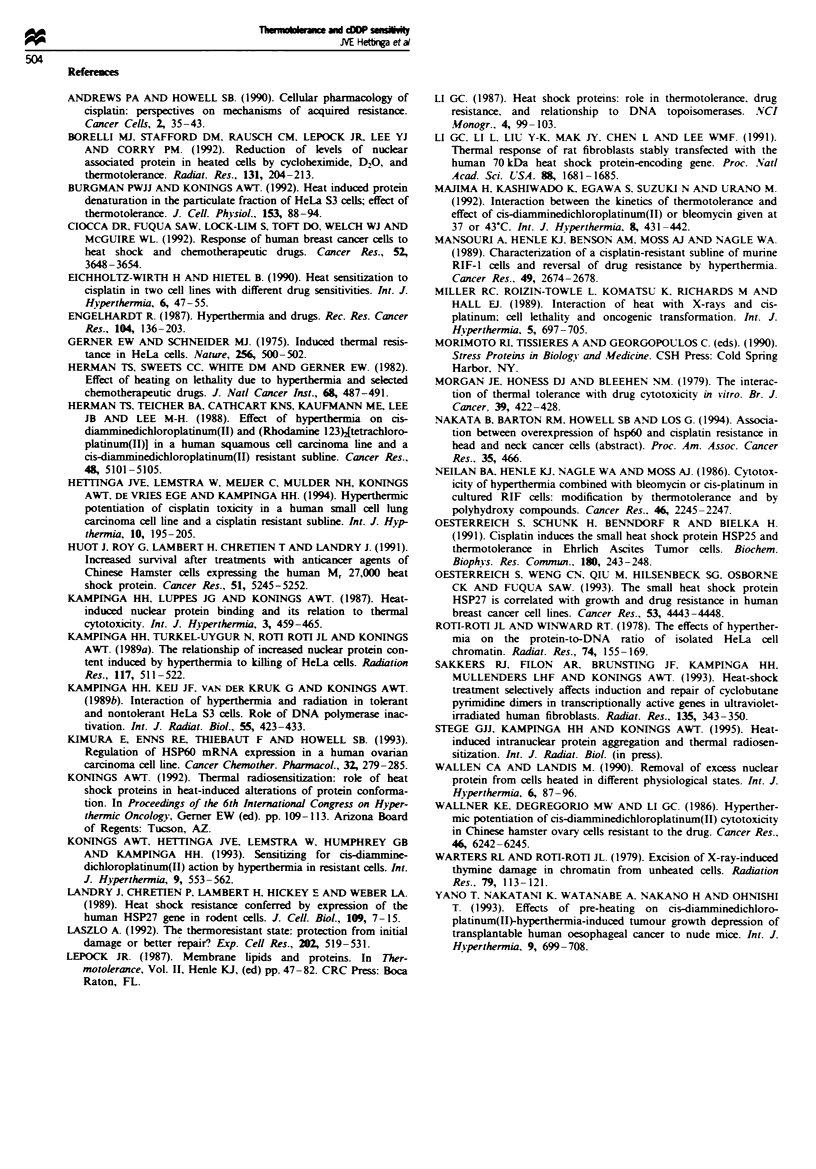# Cisplatin sensitivity and thermochemosensitisation in thermotolerant cDDP-sensitive and -resistant cell lines.

**DOI:** 10.1038/bjc.1995.100

**Published:** 1995-03

**Authors:** J. V. Hettinga, W. Lemstra, A. W. Konings, H. H. Kampinga

**Affiliations:** Department of Radiobiology, University of Groningen, The Netherlands.

## Abstract

**Images:**


British Journal of Cancer (1995) 71, 498-504

© 1995 Stockton Press All rights reserved 0007-0920/95 $9.00

### Cisplatin sensitivity and thermochemosensitisation in thermotolerant cDDP-sensitive and -resistant cell lines

JVE Hettinga, W Lemstra, AWT Konings and HH Kampinga

*Department of Radiobiology, University of Groningen, Bloemsingel 1, 9713 BZ Groningen, The Netherlands.*

**Summary** Development of thermotolerance is an important phenomenon that must be considered when thermochemotherapy with multiple heat treatments is used clinically. To study the effect of thermotolerance on cellular cisplatin (cDDP) sensitivity at 37°C and 43°C in cell lines with different cDDP sensitivities, two Ehrlich ascites tumour cell lines (one with high cDDP sensitivity and one with *in vitro* acquired cDDP resistance) were used. The results indicate that in both cell lines the state of thermotolerance *per se* did not affect the cDDP sensitivity at 37°C. Thus, general elevations in 'all' heat shock protein levels as found in thermotolerant cells apparently do not influence cDDP sensitivity to a considerable extent. The sensitising effect of a (second) heat treatment given simultaneously with a cDDP treatment was less in thermotolerant cells. Thermal enhancement ratios (TERs) at the 10% survival level for heat doses of 43°C for 30 min or 43°C for 60 min were reduced by a factor of 1.6 and 2.1 in cDDP-resistant and -sensitive thermotolerant cells respectively, as compared with control cells. Thus, protection against heat damage in thermotolerant cells seems to be paralleled by diminished thermal chemosensitisation. Although the effect of thermotolerance on the cDDP-sensitising effect was less pronounced in the resistant cells, a modifying effect on the resistance factor was not achieved.

**Keywords:** thermotolerance; cisplatin; hyperthermia; thermo-chemosensitisation; drug resistance

Development of resistance to cisplatin [cDPP, *cis*-diammine-dichloroplatinum(II)] is a major limitation to the clinical success of the drug. The subject of cDDP resistance has been extensively studied *in vitro* (reviewed by Andrews and Howell, 1990), and is most likely a multifactorial phenomenon. Decreased cellular drug accumulation, enhanced drug detoxification by glutathione or metallothioneins, reduced DNA damage induction and elevated DNA repair capacity are among the mechanisms reported to contribute to decreased sensitivity in cDDP-resistant cell lines.

Hyperthermia has been shown to enhance the cytotoxic action of a number of chemotherapeutic drugs (reviewed by Engelhardt, 1987), including cDDP. Since hyperthermia has the potential to interfere with all the above-mentioned mechanisms that can cause cDDP resistance, it may also be a suitable modality to interfere with acquired resistance. The studies performed so far on hyperthermic cDDP sensitisation in cDDP-resistant cell lines seem to indicate that, at least with higher temperatures (i.e. 43°C), drug resistance may be overcome partially (Wallner *et al.*, 1986; Herman *et al.*, 1988; Mansouri *et al.*, 1989; Konings *et al.*, 1993; Hettinga *et al.*, 1994).

For the clinical use of thermochemotherapy, optimal treatment schedules of heat and the chemotherapeutic agent must be determined. If fractionated treatments are given, the development of thermotolerance is an important phenomenon to be considered. Thermotolerance, first described by Gerner and Schneider (1975), is the transient resistant state of cells to a (second) heat treatment after being pre-exposed to hyperthermia. For successful use of thermochemotherapy it is important to know whether the thermotolerant state influences the cDDP sensitivity of cells at 37°C. In thermotolerant cells elevated levels of heat shock proteins (HSPs) have been found. These HSPs seem to play an important role in thermal resistance (for review see Morimoto *et al.*, 1990). Transfection studies with HSPs show that transfectants have increased resistance to heat (Landry *et al.*, 1989; Li *et al.*, 1991). HSPs may also influence sensitivity to cytostatic drugs. Transfectants with the human small heat shock protein HSP27 have been shown to exhibit a multidrug-resistant phenotype (Huot *et al.*, 1991) that is not P-glycoprotein mediated. Also, HSP70-overexpressing mutant Chinese hamster fibroblast cells have been found to be resistant to VM26 (Li, 1987). Thus, thermotolerant cells may also have altered drug sensitivity owing to increased levels of HSPs. Finally, in addition to being resistant to the toxic effects of heat alone, thermotolerant cells may also be less susceptible to thermochemosensitisation. A few studies comparing hyperthermic cDDP sensitisation in control and thermotolerant cells have been published. Herman *et al.* (1982) showed that induction of chronic thermotolerance by altering the heating rate led to less thermal enhancement of cDDP toxicity. Neilan *et al.* (1986), however, found that induction of acute thermotolerance did not affect the interaction of cDDP and 43°C temperature. Also, cDDP sensitivity at 37°C was unchanged. Recently Majima *et al.* (1992) also found unaltered cDDP sensitivity at 37°C when cells developed thermotolerance. Cell killing by the cDDP-hyperthermia combination treatment was shown to be lower in thermotolerant cells. Since no corrections were made for the killing effect of heat alone, it is hard to distinguish whether the reduced efficacy of the combined treatment is due to reduced heat killing in the thermotolerant cells or/and decreased thermal enhancement. Thus, the effect of thermotolerance on hyperthermic drug sensitisation is not unequivocally clear. Also, no information exists on whether thermotolerance effects may differ for cells with acquired cDDP resistance compared with the parent cells. Therefore, in the present study the effect of thermotolerance on cDDP sensitivity at 37°C, and on the sensitising effect of a (second) hyperthermia treatment at 43°C, was determined in cDDP-sensitive (EN) and -resistant (ER) Ehrlich ascites tumour cells.

### Materials and methods

### *Materials*

cDDP (Aldrich, Milwaukee, WI, USA) was stored as a stock solution of 1000 $\mu g\ ml^{-1}$ in water at $-80$°C, in portions of 1 ml in Eppendorf tubes, for at most 1 month. Tissue culture medium (RPMI-1640), fetal calf serum and newborn calf serum for the soft agar plates were obtained from Gibco (Paisley, UK). All other chemicals were purchased from Sigma (St Louis, MO, USA) or Merck (Darmstadt, Germany).

Correspondence: JVE Hettinga
Received 17 June 1994; revised 30 August 1994; accepted 3 November 1994

*Cell lines*

A cloned cDDP-sensitive Ehrlich ascites tumour (EAT) cell line (EN) and a cloned EAT cell line with *in vitro* acquired cDDP resistance (ER) were used. The ER cell line was developed by culturing EAT parent cells in the presence of 5 ng ml$^{-1}$ cDDP for 4 months, after which these cells were treated with 8 $\mu$g ml$^{-1}$ cDDP for 90 min. The surviving cells were cloned by repeated plating on soft agar. At the 10% survival level ER cells were 4.8 times more resistant to cDDP than EN cells (Konings *et al.*, 1993). Both cell lines were grown in suspension culture in RPMI-1640 medium supplemented with 10% fetal calf serum, 100 units ml$^{-1}$ penicillin and 100 $\mu$g ml$^{-1}$ streptomycin. The doubling time of the cells was about 11 h. To ensure a stable level of resistance, the ER cell line was cultured for no longer than 4 months, after which period new cells were thawed from liquid nitrogen storage.

*Hyperthermia and cisplatin treatments*

Hyperthermia was performed in precision waterbaths ($\pm$ 0.05°C) under continuous gentle shaking. One millilitre of cell suspension with a cell concentration of $1 \times 10^6$ ml$^{-1}$ was incubated in plastic tubes for various times.

To induce thermotolerance, cell suspensions with a cell concentration of $1 \times 10^6$ ml$^{-1}$ were treated for 10 min at 44°C, after which they were placed in a shaking incubator at 37°C for various times to allow development of thermotolerance before giving a second treatment with heat and/or cDDP.

For cDDP treatment, a cDDP stock sample was thawed to room temperature immediately before each experiment and diluted in complete medium. A 0.1 ml volume of a 10 × concentrated cDDP solution was added to 0.9 ml of cell suspension ($1.1 \times 10^6$ cells ml$^{-1}$). Total incubation time with cDDP was always 90 min.

For simultaneous treatments of hyperthermia and cDDP, the heat treatment was given during the first part of the incubation period with cDDP, after which the remaining incubation was performed in a 37°C waterbath.

*Clonogenic survival assay*

After treatment with hyperthermia and/or cDDP, the samples were washed once with 10 ml of medium (RPMI with 10% fetal calf serum), appropriately diluted to obtain about 100 colonies per plate, and plated on 0.5% soft agar plates with RPMI-1640, supplemented with 15% newborn calf serum and penicillin and streptomycin. The cells were mixed with $1 \times 10^5$ feeder cells [a mixture of supralethally (>200 Gy) irradiated EN and ER cells] per plate. The plates were incubated at 37°C in a carbon dioxide incubator for 7 days. Colonies containing more than 50 cells were counted. The plating efficiency was always over 90%. From the cDDP survival curves (corrected for heat kill) of each experiment the concentration needed to kill 90% of the cells ($IC_{90}$) was determined by graphical interpolation. From this, thermal enhancement ratios (TERs) were determined by dividing the $IC_{90}$ for cDDP treatment at 37°C by the $IC_{90}$ for cDDP treatment combined with hyperthermia.

*Statistical analysis*

Significance levels were calculated using the unpaired Student's *t*-test. $P < 0.05$ was considered statistically significant.

*Western blotting*

Thermotolerance was induced as described above. After various time intervals at 37°C after the thermotolerance trigger, samples were taken from the cell suspension and washed three times with phosphate-buffered saline (PBS). For every $2 \times 10^6$ cells, 20 $\mu$l of TNMP [10 mM Tris, 5 mM magnesium chloride, 10 mM sodium chloride, 0.1 mM phenyl methyl sulphonylfluoride (PMSF)] and 5 $\mu$l of DNAse I (5 mg/ml) was added to the cell pellet. After overnight incubation at 4°C an equal volume of 2 × sample buffer [100 mM Tris pH 6.8, 20% glycerol, 10% mercaptoethanol, 4% sodium dodecyl sulphate (SDS), 0.05% bromphenol blue] was added and 15-$\mu$l samples were run on 10% polyacrylamide gels. The proteins were electrophoretically transferred to nitrocellulose filters and probed with SPA 810 antibody to detect HSP72 and SPA 801 antibody to detect HSP25 (murine equivalent of human HSP27) (Stressgen, Victoria, Canada).

### Results

*Thermotolerance development in EN and ER cells*

Figure 1 shows the development of thermotolerance in EN (Figure 1a) and ER (Figure 1b) cells after a 10 min at 44°C trigger. The sensitivity to 43°C of previously unheated cells and of thermotolerant cells 1, 3, 5 and 8 h after the thermotolerance-inducing heat treatment is shown. As shown before (Konings *et al.*, 1993), the cDDP-resistant (ER) cells were less heat sensitive than the EN cells. In Figure 1c the development of thermotolerance is shown by plotting thermotolerance ratios at 50% cell kill ($TTR_{50}$) as a function of the time after the first heat treatment. The $TTR_{50}$ is the ratio of the heating time required to kill 50% of thermotolerant cells and 50% of previously unheated cells. The thermotolerant state develops rapidly and remains at least up to 8 h after the triggering dose. Figure 2 shows Western analyses of HSP25 and HSP72 levels in EN and ER cells during TT development. In control cells no HSP25 and HSP72 was detectable. Expression of both HSPs was clearly induced in both cell lines 3 h after 10 min at 44°C. Up to at least 8 h after TT induction, very high levels of both HSPs were observed in both cell lines.

Since at 5 h after 10 min 44°C both cell lines have developed a substantial level of thermotolerance which is about the same, this time point was used for further studies.

*Cisplatin sensitivity (37°C) of control and thermotolerant EN and ER cells*

In Figure 3 the cDDP sensitivity at 37°C of the EN and ER control and thermotolerant cells (5 h after thermotolerance induction) is shown. At the 10% survival level the control ER cells are 4.8 times as resistant to the drug as the control EN cells. The thermotolerant cells of both cell lines were more sensitive to cDDP treatment at 37°C than the control cells.

To determine whether this increased sensitivity was due to the state of thermotolerance or to a persisting sensitising effect of the 10 min at 44°C treatment to trigger thermotolerance, the cDDP sensitivity was investigated by separating the trigger heat dose (10 min at 44°C) and the cDDP treatment by 0–8 h incubation at 37°C. Figure 4a and 4b shows the cDDP survival curves of EN and ER cells previously unheated or treated with 10 min at 44°C during cDDP treatment or 3, 5 or 8 h before cDDP treatment. Heating during the drug treatment gives maximal enhancement of cDDP-induced cell kill. When heat and drug treatment are separated by 3 and 5 h, considerable sensitisation is still found. Eight hours after the heat treatment, however, no (ER) or almost no (EN) sensitising effect is left. The observed effect is clearly shown in Figure 4c: the sensitising effect, expressed as the thermal enhancement ratio (TER) at 10% survival, (the ratio between the cDDP dose needed to kill 90% of the cells at 37°C and the cDDP dose killing 90% of the cells when combined with hyperthermia), of the 10 min at 44°C thermotolerance triggering dose decreases with increasing interval times at 37°C before the cDDP treatment. This decay is somewhat more rapid in the ER cells than in the EN cells. Notably, 8 h after 10 min at 44°C considerable levels of thermotolerance are still observed in both cell lines (Figure 1c), and HSP25/72 levels are still high (Figure 2), whereas at this time point no (ER) or nearly no (EN) altered cDDP sensitivity is seen any more (Figure 4c). Therefore, it is unlikely that the thermotolerant status of the cells influences the cDDP sensitivity (at 37°C) directly. Furthermore, this indicates that a general elevation of HSP levels in thermotolerant cells (as shown in Figure 2) does not appear to affect the cells' sensitivity to cDDP to a great extent.

**Figure 1** Typical experiment showing thermotolerance development in EN and ER cells. The 43°C heat sensitivity of EN (**a**) and ER (**b**) cells without pretreatment (Δ) or 1 (▲), 3 (∇), 5 (▼) or 8 (□) h after 10 min at 44°C pretreatment is depicted. Three to six plates were used per data point. Bars (s.d.) are shown when they exceed the symbol. Survival curves are corrected for the cell killing effect of the thermotolerance trigger. Survival after 10 min at 44°C alone was 71.5% ± 7.6% for EN and 91.0% ± 10.2% for ER cells. (c) Thermotolerance ratios at the 50% survival level ($TTR_{50}$) as a function of time at 37°C after thermotolerance trigger (10 min at 44°C).

*Hyperthermic cDDP sensitisation in control and thermotolerant EN and ER cells*

Figure 5 shows the cDDP-sensitising effect of different heat treatments (15 min at 44°C, 30 min at 43°C, 60 min at 42°C) with about the same level of cell kill within one cell line. As can be seen, these different time–temperature combinations have, in parallel with the same cell killing effect, the same cDDP-sensitising effect. This was found to be the case in both the EN (Figure 5a) and ER cells (Figure 5b). Thus, these data are suggestive of a correlation between heat killing and thermal drug sensitisation. This would imply that, if the killing effect of a certain heat treatment is decreased, for instance by induction of thermotolerance, the sensitising effect would be expected to be lower too. To investigate this, both control and thermotolerant cells were treated with cDDP at 37°C and with cDDP combined with 30 and 60 min

**Figure 2** Western blots showing induction of HSP25 (**a**) and HSP72 (**b**) expression in EN and ER cells after 10 min at 44°C treatment. Equal amounts of cells were loaded on the gels. C0, C8, non-heated control cells 0 and 8 h after replacing the medium; TT1, TT3, TT5, TT8, thermotolerant cells 1, 3, 5 and 8 h after thermotolerance trigger.

**Figure 3** cDDP sensitivity of EN and ER control and thermotolerant cells 5 h after thermotolerance trigger. Survival curves are corrected for the cell killing effect of the thermotolerance trigger. The mean values of at least three independent experiments are given; bars (s.e.m.) are shown when they exceed the symbol.

**Figure 4** Typical experiment showing the cDDP-sensitising effect of 10 min 44°C in EN and ER cells. cDDP survival curves of EN (**a**) and ER (**b**) cells without pretreatment (Δ), after simultaneous 10 min at 44°C treatment (▲) and after 10 min 44°C pretreatment 3 (▽), 5 (▼) or 8 (□) h before cDDP treatment are depicted. Three to six plates were used per data point. Bars (s.d.) are shown when they exceed the symbol. Survival curves are corrected for the cell killing effect of the thermotolerance trigger. (**c**) The sensitising effect of 10 min at 44°C expressed as the thermal enhancement ratio (10% survival) as a function of the time between heat and cDDP treatments.

**Figure 5** cDDP sensitisation by different time–temperature combinations with the same cell killing effect in EN (**5a**) and ER (**5b**) cells. Survival surves are corrected for the cell killing effect of the heat treatments. The mean values of at least three independent experiments are given; bars (s.e.m.) are shown when they exceed the symbol.

at 43°C. cDDP survival curves were constructed and are shown in Figure 6. From these curves TERs of the 43°C heat treatments were calculated and are depicted in Figure 7. As can be deduced from this figure, the sensitising effect of 30 or 60 min of 43°C hyperthermia in both EN (Figure 7a) and ER (Figure 7b) thermotolerant cells was found to be decreased significantly by a factor of 2.1 and 1.6 respectively.

### Discussion

*cDDP sensitivity at 37°C of thermotolerant cells*

It is known (Wallner *et al*., 1986; Eichholtz-Wirth and Hietel, 1990) that when combined treatments of heat and cDDP are given, maximal potentiation is observed when both modalities are given simultaneously; the interaction gradually decreases to the additive level when heat and cDDP are separated by incubation at 37°C. This was also observed in the present study (Figure 4c). At 8 h after treatment for 10 min at 44°C, almost no enhancement of cDDP toxicity

**Figure 6** Full cDDP survival curves showing the cDDP-sensitising effect of 30 or 60 min at 43°C in EN (**a** and **b**) and ER (**c** and **d**) control (**a** and **c**) and thermotolerant (**b** and **d**) cells (5 h after thermotolerance trigger). Survival curves are corrected for the cell killing effect of the heat treatment. Mean values of at least three independent experiments are given; bars (s.e.m.) are shown when they exceed the symbol.

was observed any more. At this time the cells have become quite resistant to heat toxicity (Figure 1c) because of thermotolerance development. Since the cDDP sensitivity of the cells is equal to the sensitivity of control cells at this time point, the state of thermotolerance does not seem to confer resistance against cDDP toxicity to the cells.

Unaltered cDDP sensitivity at 37°C in thermotolerant cells is in accordance with previously published data of both *in vitro* (Neilan *et al.*, 1986; Miller *et al.*, 1989; Majima *et al.*, 1992) and *in vivo* (Yano *et al.*, 1993) studies. Also, absence of interaction of thermotolerance with the cytotoxicity of several other drugs was observed, e.g. bleomycin toxicity has been shown to be unaltered in thermotolerant cells in a number of studies (Morgan *et al.*, 1979; Neilan *et al.*, 1986; Majima *et al.*, 1992). Morgan *et al.* (1979) showed the same for BCNU toxicity. For topoisomerase II inhibitors, however, a different picture emerges from the literature: cells made thermotolerant by prior heating have been found to be resistant to several topoisomerase drugs such as VM26 (Li *et al.*, 1987), doxorubicin (Ciocca *et al.*, 1992; H.H. Kampinga, unpublished results), m-AMSA and VP16 (H.H. Kampinga, unpublished results). Conversely VP16 and VM26 have been shown to be able to induce heat shock proteins and thermotolerance (Li, 1987). For doxorubicin a direct role of HSP27 in resistance has been suggested. Overexpression of HSP27 by transfection leads to doxorubicin resistance in both hamster (Huot *et al.*, 1991) and human (Oesterreich *et al.*, 1993) cells. The level of HSP27 overexpression is correlated with the level of doxorubicin resistance. Also for cDDP evidence exists for a possible link between HSPs and resistance. cDDP has also been shown to be able to induce HSPs (Oesterreich *et al.*, 1991; authors' unpublished data). cDDP pretreatment can even induce (low) levels of thermotolerance (Oesterreich *et al.*, 1991). Also, a role of overexpression of HSP60 in cDDP resistance has been suggested (Kimura *et al.*, 1993; Nakata *et al.*, 1994). Nevertheless, the absence of a thermotolerance effect on cDDP sensitivity at 37°C may indicate that the general elevated expression of HSPs does not affect cDDP sensitivity. However, our data do not fully exclude the possibility that two counteracting processes are taking place, i.e. (decay of) thermal enhancement of cDDP action by the thermotolerance triggering heat dose and (development of) resistance to cDDP in parallel to resistance to heat (related to the accumulation of HSPs). Yet, when cDDP sensitivity of, for example, the ER cells is considered, it is clear that the same level of thermotolerance is found 5 and 8 h after the triggering heat dose. At the 5 h time point the cells are still somewhat more cDDP sensitive; no alteration in cDDP sensitivity is found at the 8 h time point. If two opposing effects were taking place, some resistance to cDDP would be expected at the 8 h time point.

So, in general it must be concluded that the mechanisms that protect thermotolerant cells against heat killing do not protect the cells against killing by most cytostatic drugs.

**Figure 7** cDDP-sensitising effect of 30 and 60 min at 43°C in EN (**a**) and ER (**b**) control and thermotolerant cells (5 h after thermotolerance induction) expressed as thermal enhancement ratio at 10% survival. The mean values of at least three independent experiments are given; bars (SEM) are shown when they exceed the symbol. (*$P<0.05$, **$P<0.005$).

Certainly a *general* elevation of HSPs by inducing thermotolerance does not seem to protect cells against cDDP toxicity to a considerable extent. Yet it can also be speculated that different HSPs may have opposing influences on cDDP toxicity; therefore a role for specific HSPs in cDDP sensitivity and resistance cannot be excluded.

### *cDDP sensitisation by hyperthermia in thermotolerant cells*

The cDDP-sensitising effect of 43°C hyperthermia was shown in the present study to be diminished in thermotolerant cells. This is in accordance with previously published studies by Herman *et al.* (1982) and Majima *et al.* (1992). On the other hand, Neilan *et al.* (1986) found the same level of thermochemosensitisation in control and thermotolerant RIF cells. Their data, however, show very low hyperthermic enhancement of cDDP toxicity in the control cells, and therefore it might be hard to detect a putative reduction in TER in the thermotolerant cells. A recently published *in vivo* study (Yano *et al.*, 1993) is also indicative of less thermal enhancement of cDDP toxicity in thermotolerant tumours. Hyperthermic potentiation of some other drugs, such as bleomycin and BCNU, is also lower in thermotolerant cells (Morgan *et al.*, 1979; Herman *et al.*, 1982; Neilan *et al.*, 1986; Majima *et al.*, 1992).

Thus, it seems that the mechanisms responsible for hyperthermic cell killing and hyperthermic drug sensitisation must in part be the same. Heat has been shown to cause denaturation and insolubilisation of cellular proteins (Lepock, 1987; Burgman and Konings, 1992). This leads to, among other things, an increase in the protein mass of nuclei isolated from heated cells (Roti-Roti and Winward, 1978). Under several conditions, including thermotolerance, a good correlation has been found between the extent and duration of this so-called nuclear protein aggregation and thermal cell killing (Kampinga *et al.*, 1989*a*). This nuclear protein aggregation may also be one of the major mechanisms responsible for thermal potentiation of killing by a number of drugs, by hampering repair of drug-induced DNA damage. Therefore, reduced protein aggregation or accelerated disaggregation, as observed (Kampinga *et al.*, 1987, 1989*a*; Wallen and Landis, 1990; Borelli *et al.*, 1992; Laszlo, 1992) in thermotolerant cells, may not only cause less cell killing, but may also lead to less inhibition of repair of drug damage, thereby decreasing the thermal potentiation of drug action. The latter would be similar to what has been suggested for thermal radiosensitisation: chromatin alterations due to heat-induced nuclear protein aggregation reduce the repairability of damaged DNA (Warters and Roti-Roti, 1979; Sakkers *et al.*, 1993), leading to enhanced radiosensitivity (Kampinga *et al.*, 1989*b*; Konings, 1992; Stege *et al.*, 1995).

The observed reduction of TERs was significantly lower in the resistant ER cells than in the sensitive EN cells ($1.59 \pm 0.10$ *vs* $2.09 \pm 0.14$, $P<0.005$). It is also clear that TERs in control cells were higher in the cDDP-sensitive cell line than in the cDDP-resistant subline. Figure 5a shows that a saturation of cell killing occurs in the EN cell line for doses of cDDP above 1 $\mu g\ ml^{-1}$ when combined with hyperthermia. In our earlier experiments (Konings *et al.*, 1993) we used only these higher cDDP doses for the survival curves and fitted straight lines through these by linear regression. Using this method we found higher TER values for ER cells than for EN cells. Our current data (using lower cDDP doses) show that cDDP survival curves for the EN cells are not well represented by straight lines and also show that, by doing so, the TERs that can be achieved in EN cells may have been underestimated. Thus, no reduction of resistance can be obtained by the combination of 43°C hyperthermia and cDDP in these cell lines. Even though induction of thermotolerance gives less reduction of TERs in the cDDP-resistant cells, the absolute TERs are similar in thermotolerant cDDP-sensitive and -resistant cells. Therefore it must be concluded that thermotolerance has no major impact on the resistance factor.

When thermochemotherapy is given in a fractionated schedule, thermotolerance can develop. In the present study we showed that the state of thermotolerance itself does not seem to affect the sensitivity of tumour cells to cDDP treatment at 37°C to a great extent. However, the efficacy of a second heat treatment combined with drug application is greatly reduced. Both the cell killing and the drug-sensitising effect of the second heat treatment is lowered in the thermotolerant cells. Therefore, to obtain maximal effect of a heat–drug treatment it is necessary to avoid thermotolerance. When a second treatment consists of drug treatment only, thermotolerance does not hamper the efficacy of this treatment. In the present study it was also shown that the sensitising effects in thermotolerant cDDP-sensitive and -resistant cells are the same, so thermotolerance development does not influence cDDP resistance.

**Acknowledgements**

This study has been financially supported by Grant GUKC 91-09 of the Dutch Cancer Society (NKB, KWF).

**References**

ANDREWS PA AND HOWELL SB. (1990). Cellular pharmacology of cisplatin: perspectives on mechanisms of acquired resistance. *Cancer Cells*, **2**, 35-43.

BORELLI MJ, STAFFORD DM, RAUSCH CM, LEPOCK JR, LEE YJ AND CORRY PM. (1992). Reduction of levels of nuclear associated protein in heated cells by cycloheximide, $D_2O$, and thermotolerance. *Radiat. Res.*, **131**, 204-213.

BURGMAN PWJJ AND KONINGS AWT. (1992). Heat induced protein denaturation in the particulate fraction of HeLa S3 cells; effect of thermotolerance. *J. Cell. Physiol.*, **153**, 88-94.

CIOCCA DR, FUQUA SAW, LOCK-LIM S, TOFT DO, WELCH WJ AND McGUIRE WL. (1992). Response of human breast cancer cells to heat shock and chemotherapeutic drugs. *Cancer Res.*, **52**, 3648-3654.

EICHHOLTZ-WIRTH H AND HIETEL B. (1990). Heat sensitization to cisplatin in two cell lines with different drug sensitivities. *Int. J. Hyperthermia*, **6**, 47-55.

ENGELHARDT R. (1987). Hyperthermia and drugs. *Rec. Res. Cancer Res.*, **104**, 136-203.

GERNER EW AND SCHNEIDER MJ. (1975). Induced thermal resistance in HeLa cells. *Nature*, **256**, 500-502.

HERMAN TS, SWEETS CC, WHITE DM AND GERNER EW. (1982). Effect of heating on lethality due to hyperthermia and selected chemotherapeutic drugs. *J. Natl Cancer Inst.*, **68**, 487-491.

HERMAN TS, TEICHER BA, CATHCART KNS, KAUFMANN ME, LEE JB AND LEE M-H. (1988). Effect of hyperthermia on cis-diamminedichloroplatinum(II) and (Rhodamine 123)$_2$[tetrachloroplatinum(II)] in a human squamous cell carcinoma line and a cis-diamminedichloroplatinum(II) resistant subline. *Cancer Res.*, **48**, 5101-5105.

HETTINGA JVE, LEMSTRA W, MEIJER C, MULDER NH, KONINGS AWT, DE VRIES EGE AND KAMPINGA HH. (1994). Hyperthermic potentiation of cisplatin toxicity in a human small cell lung carcinoma cell line and a cisplatin resistant subline. *Int. J. Hyperthermia*, **10**, 195-205.

HUOT J, ROY G, LAMBERT H, CHRETIEN T AND LANDRY J. (1991). Increased survival after treatments with anticancer agents of Chinese Hamster cells expressing the human $M_r$ 27,000 heat shock protein. *Cancer Res.*, **51**, 5245-5252.

KAMPINGA HH, LUPPES JG AND KONINGS AWT. (1987). Heat-induced nuclear protein binding and its relation to thermal cytotoxicity. *Int. J. Hyperthermia*, **3**, 459-465.

KAMPINGA HH, TURKEL-UYGUR N, ROTI ROTI JL AND KONINGS AWT. (1989*a*). The relationship of increased nuclear protein content induced by hyperthermia to killing of HeLa cells. *Radiation Res.*, **117**, 511-522.

KAMPINGA HH, KEIJ JF, VAN DER KRUK G AND KONINGS AWT. (1989*b*). Interaction of hyperthermia and radiation in tolerant and nontolerant HeLa S3 cells. Role of DNA polymerase inactivation. *Int. J. Radiat. Biol.*, **55**, 423-433.

KIMURA E, ENNS RE, THIEBAUT F AND HOWELL SB. (1993). Regulation of HSP60 mRNA expression in a human ovarian carcinoma cell line. *Cancer Chemother. Pharmacol.*, **32**, 279-285.

KONINGS AWT. (1992). Thermal radiosensitization: role of heat shock proteins in heat-induced alterations of protein conformation. In *Proceedings of the 6th International Congress on Hyperthermic Oncology*, Gerner EW (ed). pp. 109-113. Arizona Board of Regents: Tucson, AZ.

KONINGS AWT, HETTINGA JVE, LEMSTRA W, HUMPHREY GB AND KAMPINGA HH. (1993). Sensitizing for cis-diamminedichloroplatinum(II) action by hyperthermia in resistant cells. *Int. J. Hyperthermia*, **9**, 553-562.

LANDRY J, CHRETIEN P, LAMBERT H, HICKEY E AND WEBER LA. (1989). Heat shock resistance conferred by expression of the human HSP27 gene in rodent cells. *J. Cell. Biol.*, **109**, 7-15.

LASZLO A. (1992). The thermoresistant state: protection from initial damage or better repair? *Exp. Cell Res.*, **202**, 519-531.

LEPOCK JR. (1987). Membrane lipids and proteins. In *Thermotolerance*, Vol. II, Henle KJ, (ed) pp. 47-82. CRC Press: Boca Raton, FL.

LI GC. (1987). Heat shock proteins: role in thermotolerance, drug resistance, and relationship to DNA topoisomerases. *NCI Monogr.*, **4**, 99-103.

LI GC, LI L, LIU Y-K, MAK JY, CHEN L AND LEE WMF. (1991). Thermal response of rat fibroblasts stably transfected with the human 70 kDa heat shock protein-encoding gene. *Proc. Natl Acad. Sci. USA*. **88**, 1681-1685.

MAJIMA H, KASHIWADO K, EGAWA S, SUZUKI N AND URANO M. (1992). Interaction between the kinetics of thermotolerance and effect of cis-diamminedichloroplatinum(II) or bleomycin given at 37 or 43°C. *Int. J. Hyperthermia*, **8**, 431-442.

MANSOURI A, HENLE KJ, BENSON AM, MOSS AJ AND NAGLE WA. (1989). Characterization of a cisplatin-resistant subline of murine RIF-1 cells and reversal of drug resistance by hyperthermia. *Cancer Res.*, **49**, 2674-2678.

MILLER RC, ROIZIN-TOWLE L, KOMATSU K, RICHARDS M AND HALL EJ. (1989). Interaction of heat with X-rays and cis-platinum; cell lethality and oncogenic transformation. *Int. J. Hyperthermia*, **5**, 697-705.

MORIMOTO RI, TISSIERES A AND GEORGOPOULOS C. (eds). (1990). *Stress Proteins in Biology and Medicine*. CSH Press: Cold Spring Harbor, NY.

MORGAN JE, HONESS DJ AND BLEEHEN NM. (1979). The interaction of thermal tolerance with drug cytotoxicity *in vitro*. *Br. J. Cancer*, **39**, 422-428.

NAKATA B, BARTON RM, HOWELL SB AND LOS G. (1994). Association between overexpression of hsp60 and cisplatin resistance in head and neck cancer cells (abstract). *Proc. Am. Assoc. Cancer Res.*, **35**, 466.

NEILAN BA, HENLE KJ, NAGLE WA AND MOSS AJ. (1986). Cytotoxicity of hyperthermia combined with bleomycin or cis-platinum in cultured RIF cells: modification by thermotolerance and by polyhydroxy compounds. *Cancer Res.*, **46**, 2245-2247.

OESTERREICH S, SCHUNK H, BENNDORF R AND BIELKA H. (1991). Cisplatin induces the small heat shock protein HSP25 and thermotolerance in Ehrlich Ascites Tumor cells. *Biochem. Biophys. Res. Commun.*, **180**, 243-248.

OESTERREICH S, WENG CN, QIU M, HILSENBECK SG, OSBORNE CK AND FUQUA SAW. (1993). The small heat shock protein HSP27 is correlated with growth and drug resistance in human breast cancer cell lines. *Cancer Res.*, **53**, 4443-4448.

ROTI-ROTI JL AND WINWARD RT. (1978). The effects of hyperthermia on the protein-to-DNA ratio of isolated HeLa cell chromatin. *Radiat. Res.*, **74**, 155-169.

SAKKERS RJ, FILON AR, BRUNSTING JF, KAMPINGA HH, MULLENDERS LHF AND KONINGS AWT. (1993). Heat-shock treatment selectively affects induction and repair of cyclobutane pyrimidine dimers in transcriptionally active genes in ultraviolet-irradiated human fibroblasts. *Radiat. Res.*, **135**, 343-350.

STEGE GJJ, KAMPINGA HH AND KONINGS AWT. (1995). Heat-induced intranuclear protein aggregation and thermal radiosensitization. *Int. J. Radiat. Biol.* (in press).

WALLEN CA AND LANDIS M. (1990). Removal of excess nuclear protein from cells heated in different physiological states. *Int. J. Hyperthermia*, **6**, 87-96.

WALLNER KE, DEGREGORIO MW AND LI GC. (1986). Hyperthermic potentiation of cis-diamminedichloroplatinum(II) cytotoxicity in Chinese hamster ovary cells resistant to the drug. *Cancer Res.*, **46**, 6242-6245.

WARTERS RL AND ROTI-ROTI JL. (1979). Excision of X-ray-induced thymine damage in chromatin from unheated cells. *Radiation Res.*, **79**, 113-121.

YANO T, NAKATANI K, WATANABE A, NAKANO H AND OHNISHI T. (1993). Effects of pre-heating on cis-diamminedichloroplatinum(II)-hyperthermia-induced tumour growth depression of transplantable human oesophageal cancer to nude mice. *Int. J. Hyperthermia*, **9**, 699-708.